# Clinical Evaluation of Forceps Eruption: Reestablishing Biologic Width and Restoring No Restorable Teeth

**Published:** 2006-04-01

**Authors:** Akbar Khayat, Shayan Fatehi

**Affiliations:** 1 *Department of Endodntics, School of Dentistry, Shiraz University of Medical Sciences, Shiraz, Iran*; 2 *Endodontist, Shiraz, Iran*

**Keywords:** Biologic Width, Extrusion, Forceps Eruption

## Abstract

**INTRODUCTION:** Complicated crown- root fractures, extended caries and iatrogenic destruction often result in insufficient sound tooth structures and compromise the biologic width. Two common options for re-establishing flap with osseous surgery. Although some advantages are related to these two options, but coronal movement of gingival and alveolar bone in orthodontic extrusion, esthetic problem and inconsistent topography between the involved tooth and the adjacent teeth following osseous surgery are the involved tooth and the adjacent teeth following osseous surgery are the major disadvantages of these two approaches. The purpose of this investigation was to evaluate clinically as well as radiographically the effect of surgical extrusion upon the surrounding root structures.

**MATERIALS AND METHODS:** The material consisted of 21 developed single roots (1 upper and 3 lower) surgically extruded in 17 patients (15 male and 2 female mean age 26 years, ranging 10-40). The indication for surgical extrusion was in 15 cases complicated crown root fracture and in 6 cases early loss of the crown due to an extensive decay. The roots were used where there were completed root developments and the apical fragments were long enough to accommodate a post retained crown. Preoperative radiograph as well as photograph was taken and the clinical and radiographic findings were monitored. The roots were transplanted in their socket in order to reestablish the biologic width. Fixation was carried out with a suture splint and/ or periodontal dressing for 7 days. Recall radiographs were taken at 1 and 4 weeks and at 3 month internals over a 12- month period.

**RESULTS:** Clinically none of the material of 21 teeth demonstrated ankylosis, abnormal mobility and sensibility to percussion or palpation radiographically, PDL healing at 12- month follow up was found in 20 teeth (95.2%).

**CONCLUSION:** successful results up to the time of evaluation encouraged further use of surgical extrusion. Long term evaluation is recommended.

## INTRODUCTION

To perform a coronal restoration of a tooth with a complicated crown- root fracture or in the case with an extended caries, it is often necessary to re-establish the biologic width for the placement of restorative margin. Orthodontic extrusion and apically positioned flap and osseous surgery are two commonly used options to re-establish the biologic width. Orthodontic extrusion methods were introduced in 1973 by Heithersay (1).

Although orthodontic extrusion is reported with suitable results, it has been demonstrated that this method leads to a coronal movement of the marginal gingiva and alveolar bone (2-5). Additionally, the relapse of the root following orthodontic extrusion is a common finding as a result of stretched state of marginal periodontal fibers. Many authors advocated periodontal surgery and supracrestal fiberotomy in order to remove displaced tissues, and prevent relapse of tooth intrusion (6-8).

Apically positioned flap and osseous surgery is another method to re-establish the biologic width. This technique also has limitations, and should be used where the surgery does not compromise the esthetic result (9). Forceps eruption or intra-alveolar transplantation- the other option to re-establish biologic width- was introduced in 1978 by Tegsjo et al (10) and was further developed by Buhler (11) and Kahnbery (12).

Forceps eruption is relatively simple, and enables transplantation of the root in its socket to a supragingival position, including rotation. In this method the time factor for transplantation is most critical for the success of treatment, and prognostically can be compared with immediate replantation of the teeth (13-14). In forceps eruption, the root does not leave the socket. If the root has been removed for any reason such as inspection, maximum extra oral time does not exceed two minutes, and potential deleterious effect of extra-oral time for dryness of the periodontal cells is eliminated (15). The purpose of this study was to show clinically as well as radiographically the healing of supporting root tissues after immediate replantation by forceps eruption.

## MATERIALS AND METHODS

The material included 21 single roots (18 upper and 3 lower) in 17 patients (15 male, 2 female) who referred to the department of endodontic of Shiraz University Dental School because of complicated crown- root fracture or extensive caries. The patients age ranged from 10 to 40 years (mean of 26 years). The roots needed transplantation in order to re-establish the biologic width.

To re-establish the biologic width, surgical extrusion as an alternative to orthodontic extrusion or periodontal surgery was selected and offered to the patients. After obtaining informed consent, all the cases were scored photographically.

**Figure 1:**
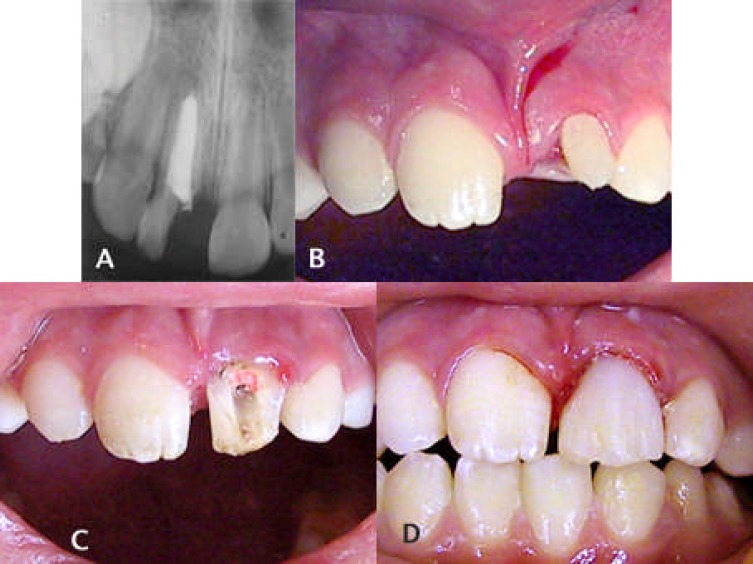
A complicated crown-root fracture on tooth 9 in a 10-year-old Afghanian boy.

Periapical radiography was carried out; using standardized parallel techniques (24×36 mm film). The roots were used where there were completed root development and the apical fragments were long enough to accommodate a post- retained crown. Endodontic treatment was performed previously or was carried out prior to forceps eruption. After anesthesia with 2% lidocaine 1:100000 epinephrine, luxation and extrusion of the roots were performed with a fine elevator after extirpation of the covering soft tissue. The extracted root was then inspected for incomplete fractures which would contraindicate the treatment plan. In the case of palatally inclined fracture, where the fracture line slanted towards the palatal or proximal sides and the level of the gingiva was more apical at the buccal side, the roots were rotated and placed to an appropriate position (Figure 1A), (Figure 1B), (Figure 1C) and (Figure 1D).

Immobilization of the roots in their new position was secured by simple interrupted interproximal sutures and / or surgical dressing.

Postoperative controls, including standard radiograph, clinical examination with probe and percussion as well as mobility tests were performed in weeks 1 and 4, months 3 and 6, up to 1 year after the surgery. Three different types of root resorption were radiographically described and recorded, as defined by Andreasen (16):

1. Surface resorption,

2. replacement resorption,

3. inflammatory resorption.

**Figure 2:**
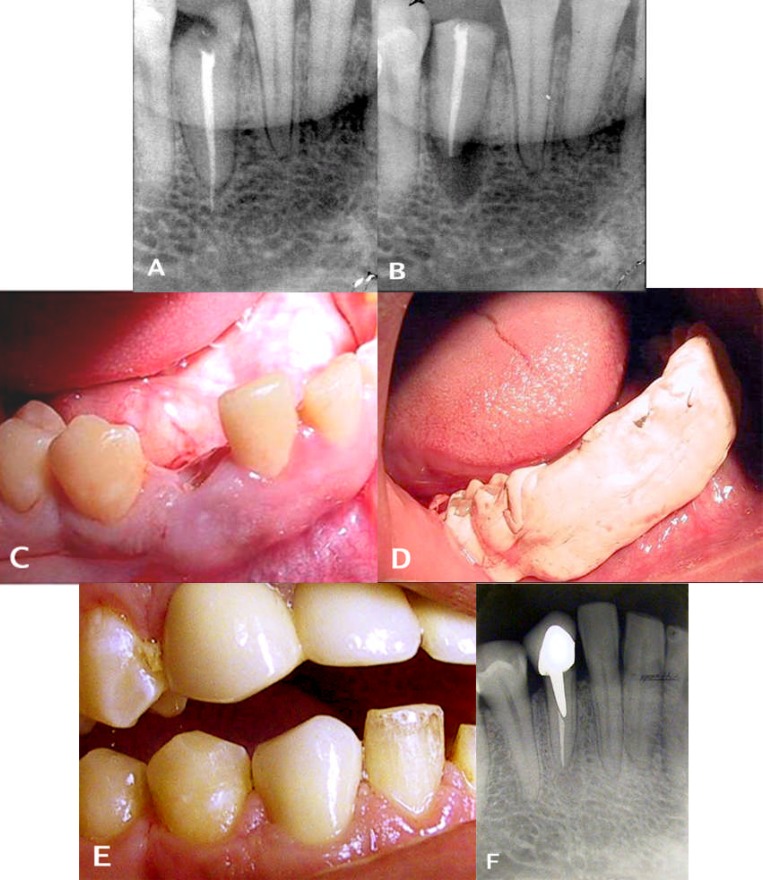
**A.** Preoperative radiograph appearance of a crown- root fracture on tooth 27.

Radiographic examination for periapical lesion, root resorption, marginal bone loss and signs of ankylosis was performed. In the clinical follow- up, a metal percussion sound indicated ankylosis, and the loosening grade was controlled by means of a mobility test based on a scale of 0-3 (grade 0: no abnormal mobility; grade 1: abnormal horizontal mobility of not more than 1 mm; grade 2: abnormal horizontal mobility of more than 1 mm; grade 3: abnormal horizontal mobility of more than one millimeter and axial mobility) (17). A completely successful result was recorded when the tooth was in function and no clinical signs of ankylosis, percussion, palpation or abnormal mobility and radiographic signs of root resorption, periapical pathology or crestal bone resorption were present. preoperative and postoperative radiographic and clinical data were monitored and compared with those taken previously (Figure 2A, Figure 2B, Figure 2C, Figure 2D, Figure 2E and Figure 2F). Only the teeth with a minimum follow-up period of 1 year were included in this study.

**A** and **B**. Radiographic and photographic of oblique crown- root fracture below the marginal bone mesial on tooth 9.

**C.** The root is rotated 90 degree (mesial aspect at the labial aspect) and transplanted.

**D.** After placement of the resin core builds up

## RESULTS

Table 1 shows supporting tissue healing related to various clinical and radiographic factors. Clinically, none of the material of 21 teeth demonstrated ankylosis, abnormal mobility and sensibility to percussion or palpation. Radiographically, PDL healing at 12-month follow- up was found in 20 teeth (95.2%). 4 teeth (19%) showed crestal bone resorption ranging between 1-3 mm.

## DISCUSSION

The result of forceps eruption so far shows that all the teeth functioned with normal mobility and no sensibility to percussion and palpation was present. Complete bone formation was also found in all cases (100%) after 6 months. This phenomenon indicated that damaged tissue left in the socket could be healed by the source of undifferentiated cells, careful tissue handling, immobilization of the root and wound edges closer which are all factors to reduce the risk of infection. PDL healing was demonstrated in 95% of the roots. This finding is paralleled by the findings following immediate replantation of avulsed teeth in which 85-97% rate of PDL healing is demonstrated (18).

In this study, none of the cases demonstrated ankylosis. This good result is predictable after stabilization with a periodontal dressing and/or suture splint and is parallel with other findings (19) which indicated that auto transplantation following fixation with a suture splint for a week was more successful than with an extended and rigid fixation.

In the present study, the results are in agreement with a similar study evaluating the same parameters following surgical extrusion, performed by Tegsjo et al (20) and Kahnberg (21), in which PDL healing with no or slight root resorption (surface resorption) had been year mean observation time follow-up, a high occurrence of periapical healing (95%) and regeneration of PDL was found (74.3%).

**Table 1:** Clinical and radiographic signs of supporting tissue healing at different interval times

	**Observation period (month)**	**1**	**3**	**6**	**12**
**Clinical and radiographic signs (n=21)**	Mobility	-	-	-	One grade 1
	Ankylosis	-	-	-	-
	Percussion	-	-	-	-
	Palpation	-	-	-	-
	Complate bone formation	10	16	21	21
	PDL healing	10	15	20	20
	Crestal bone resorption	3	4	4	4

Crestal bone resorption was seen radiographically in 4 roots, and clinically a pathological pocket was found in only one instance. This phenomenon was seen at the mesial corner and probably was due to pressure applied by the elevator.

In conclusion, we observed that a careful immediate forceps eruption had many advantages and could be considered as an alternative to extraction. Long-term evaluations are suggested to be performed.
